# Supplementary material for: Structure of the Methanosarcina mazei Mtr complex bound to the oxygen-stress responsive small protein MtrI
Source: Nat Commun. 2025 Dec 23;17:133. doi: 10.1038/s41467-025-67705-5 (PMC12774963; doi:10.1038/s41467-025-67705-5)
Supplement: Supplementary file 1 — Supplementary Information [file 41467_2025_67705_MOESM1_ESM.pdf]

Structure of the Methanosarcina mazei Mtr complex bound to the oxygen-stress responsive small protein MtrI - Supplementary information

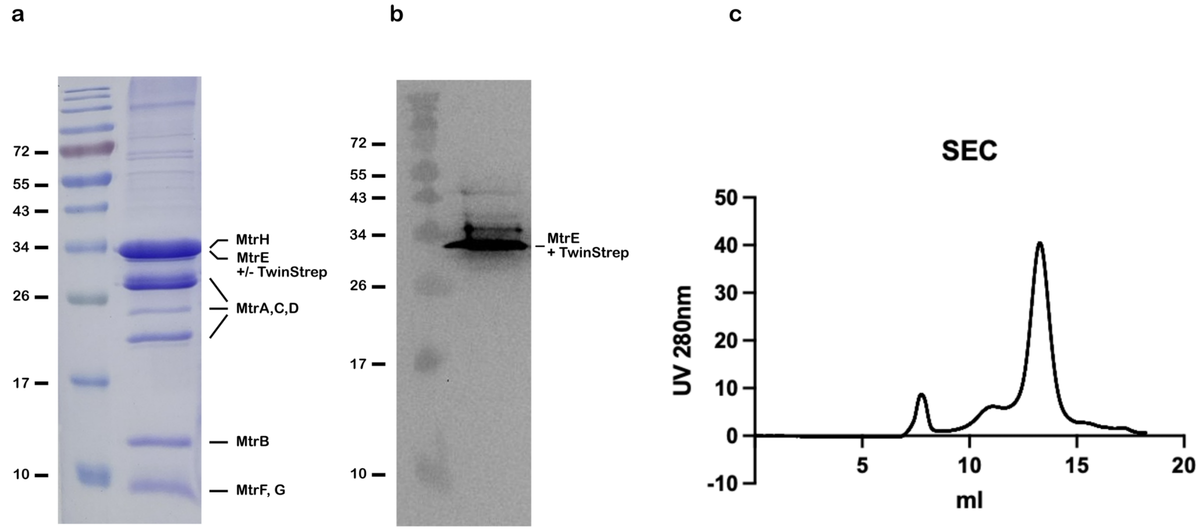

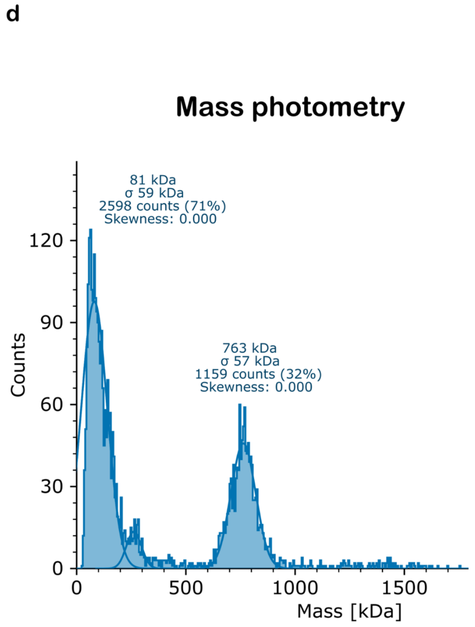

e

#	Accession	Description	Score	Coverage	# Peptides	# PSM
1	P80650	MtrH	3028,63	80,38%	31	1740
2	Q03001	Dystonin OS=Homo sapiens	0	65,68%	568	1360
3	O59640	**MtrA**	1117,75	89,17%	24	868
4	P11532	Dystrophin OS=Homo sapiens	0	71,45%	310	746
5	P15924	Desmoplakin OS=Homo sapiens	17,95	77,99%	327	741
6	P80651	MtrE	694,16	21,71%	10	670
7	Q8PW00	Rubrerythrin OS=Methanosarcina mazei	702,46	95,40%	36	591
8	P04264	Keratin, type II cytoskeletal 1 OS=Homo sapiens	507,69	75,62%	75	560
9	P80655	MtrB	1076,19	57,41%	7	553
10	Q07283	Trichohyalin OS=Homo sapiens	0	80,44%	255	551
11	P80656	MtrG	1087,48	98,61%	12	545
12	Q60186	V-type ATP synthase alpha chain OS=Methanosarcina mazei	306,62	75,43%	46	516
47	Q8PW06	60 kDa chaperonin OS=Methanosarcina mazei	149,03	87,31%	52	195
48	P80654	MtrF	355,66	50,00%	5	191
49	Q8PTW8	RAP domain-containing protein OS=Methanosarcina mazei	0	50,84%	101	187
594	Q8PXC3	Probable RNA 2'-phosphotransferase OS=Methanosarcina mazei	5,87	63,29%	19	40
595	O59638	MtrC	44,47	12,36%	4	40
596	Q8PSK9	PPK2 domain-containing protein OS=Methanosarcina mazei	0	57,35%	20	40
996	Q8PT68	ABC transporter, permease protein OS=Methanosarcina mazei	0	20,25%	10	16
997	P80653	MtrD	9,07	4,00%	2	16

Supplementary Fig. 1. Mtr-purification and protein analysis. a, 15% SDS-gel of concentrated StrepTactin eluate. **b**, SDS-gel corresponding anti-TwinStrep-tag antibody western blot. **c**, Size-exclusion chromatography of DDM-solubilized Mtr complex using a Superose 6 Increase 10/300 **GL** column. Elution at 13.3 ml

corresponds to a complex size as previously reported for Mtr. **d**, mass photometry of LMNG-solubilized and successively detergent-freed Mtr complex. **e**, mass spectrometry results of LMNG-solubilized and purified Mtr complex, analyzed with ProteomeDiscoverer.

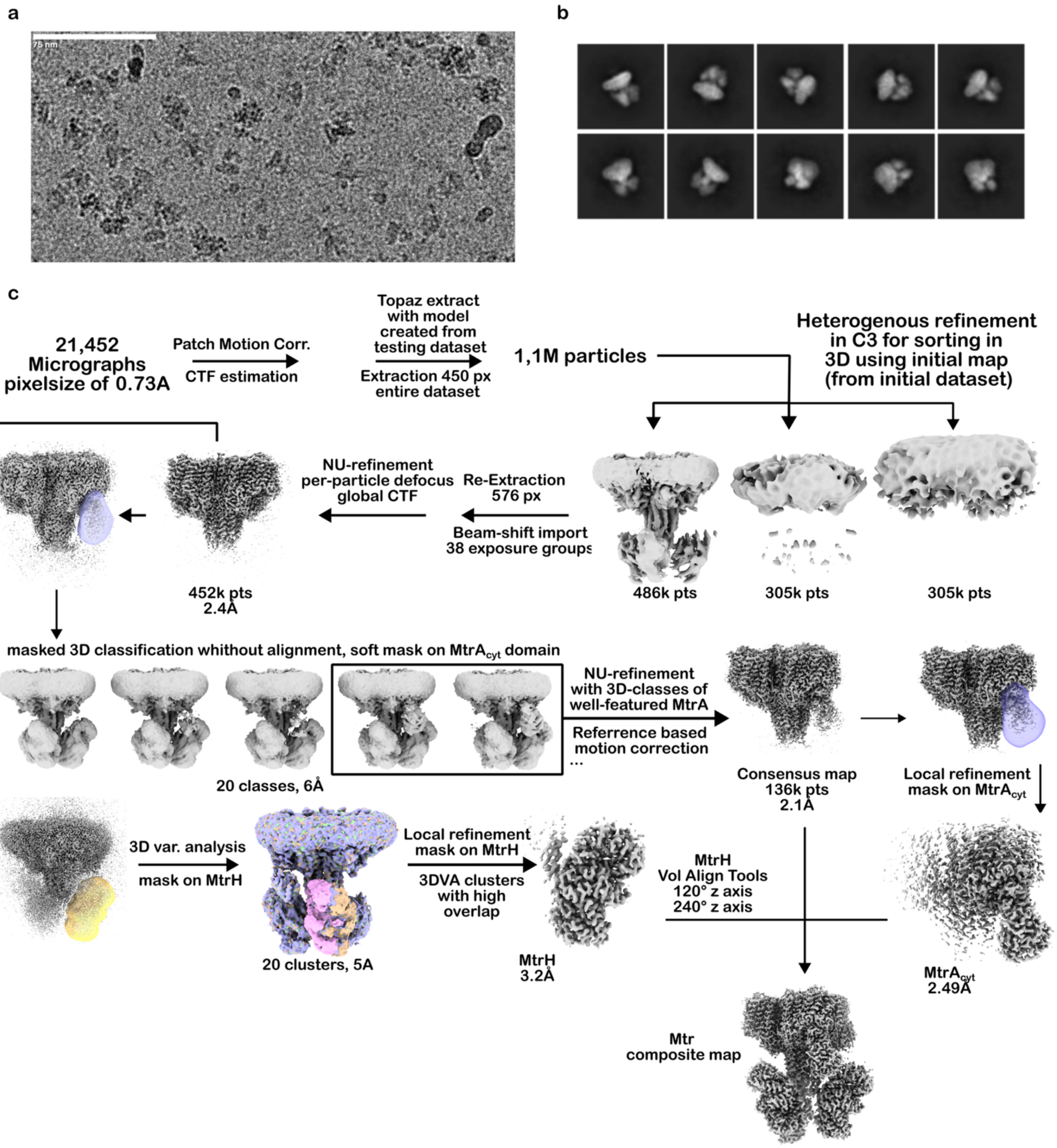

Supplementary Fig. 2. CryoEM data processing workflow. a, Exemplary micrograph with 75 nm scale bar. **b**, Selected 2D class averages depicting diverse particle orientations. **c**, Mtr data processing tree

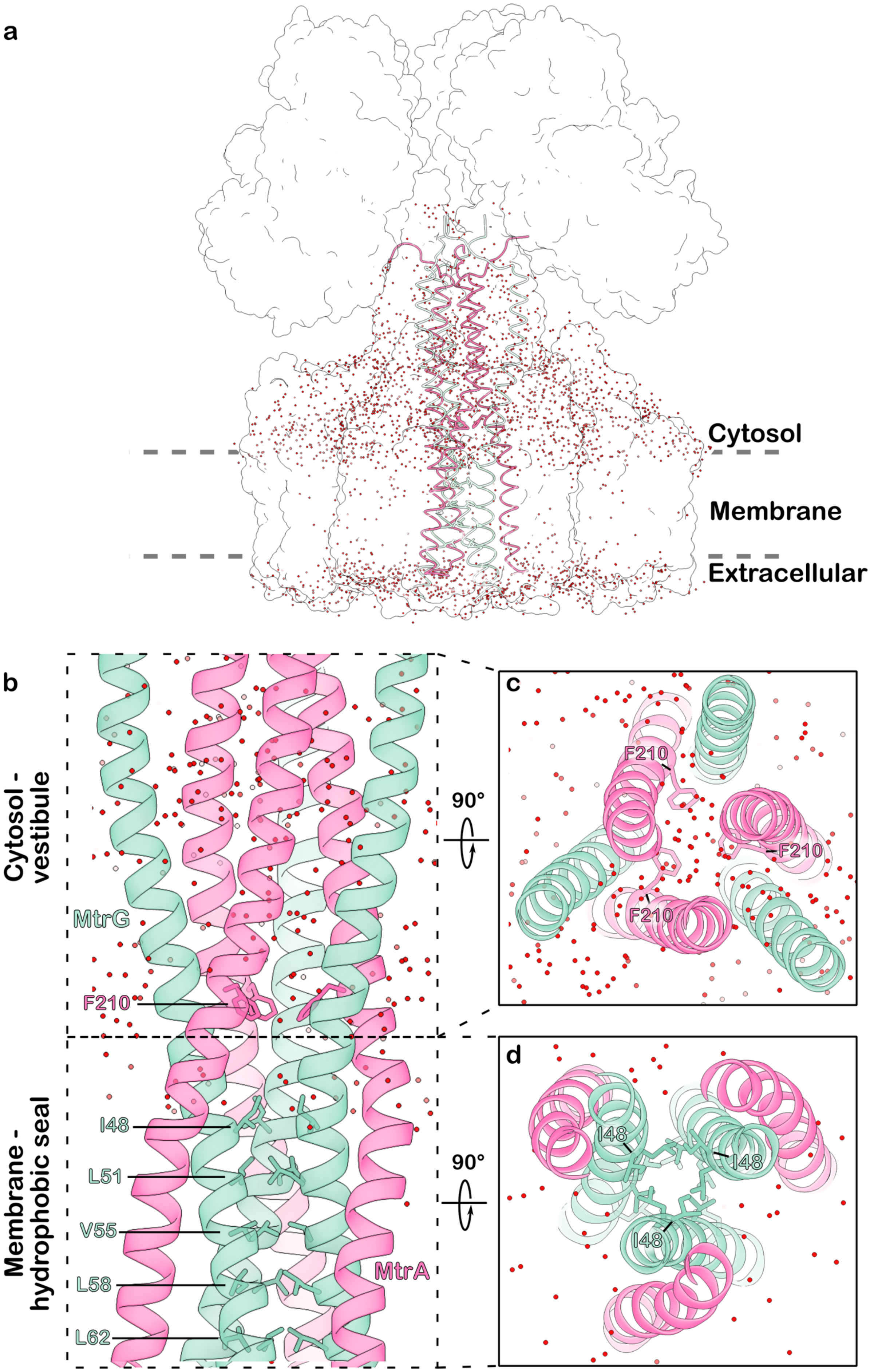

a
Cytosol
Membrane
Extracellular
b
Cytosol - vestibule
MtrG
F210
Membrane - hydrophobic seal
I48
L51
V55
L58
L62
MtrA
90°
90°
c
F210
F210
F210
d
I48
I48
I48

Supplementary Fig. 3. MtrA and MtrG form the core of the central stalk. a, overview of water molecules within the Mtr and solvation shell predicted using *douse* (PHENIX). **b**, Side view of the central stalk showing the water-filled, cytosolic vestibule formed by MtrA and the hydrophobic seal formed by MtrG within the membrane. **c**, **d**, top views from the cytosolic side toward the membrane, highlighting the water-filled vestibule (c) and the hydrophobic seal devoid of water molecules (d).

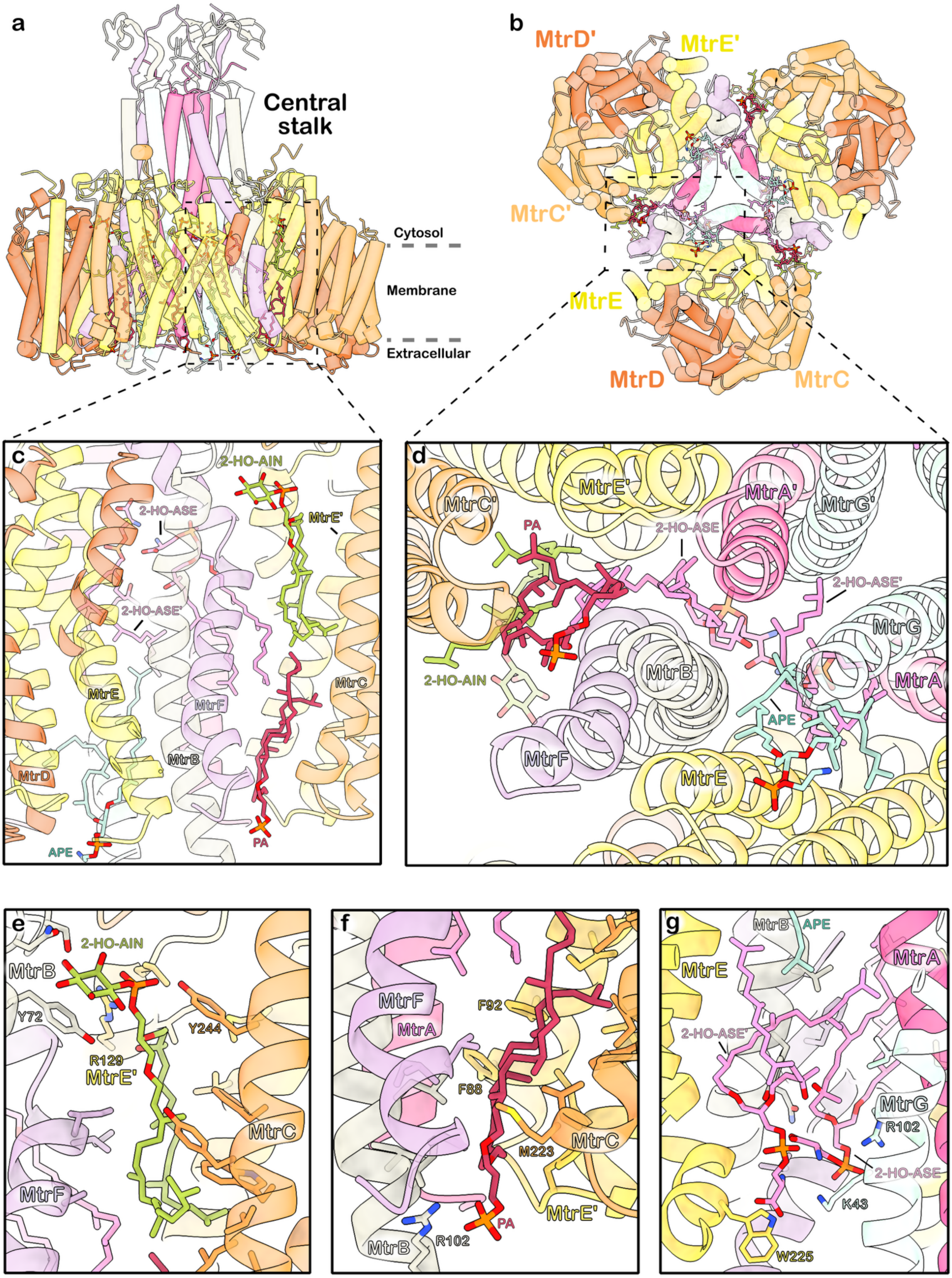

a
Central stalk
Cytosol
Membrane
Extracellular
b
MtrD'
MtrE'
MtrC'
MtrE
MtrD
MtrC
c
2-HO-AIN
2-HO-ASE
MtrE'
2-HO-ASE'
MtrE
MtrF
MtrC
MtrB
MtrD
APE
PA
d
MtrC'
MtrE'
MtrA'
MtrG'
PA
2-HO-ASE
2-HO-ASE'
MtrG
2-HO-AIN
MtrB
MtrA
APE
MtrF
MtrE
e
2-HO-AIN
MtrB
Y72
Y244
R129
MtrE'
MtrC
MtrF
f
MtrF
MtrA
F92
F88
M223
MtrC
MtrE'
PA
MtrB
R102
g
MtrB
APE
MtrE
MtrA
2-HO-ASE'
MtrG
R102
2-HO-ASE
K43
W225

Supplementary Fig. 4. Archaeal etherlipids are embedded within the membrane subunits mediating subunit interaction. a, **b,** Side and bottom views of the Mtr complex shown in transparent cartoon representation highlighting the overall lipid distribution. **c**, **d**, Zoom-in views of one of the three lipid planes formed by five lipids, detailing specific protein–lipid interactions from side and bottom perspectives. **e**, Close-up view of the 2-hydroxy-archaeolphosphatidylinositol (2-HO-AIN) binding site. **f**, Close-up view of the archaeolphosphate (PA) binding site. **g**, Close-up view of the 2-hydroxy-archaeolphosphatidylserine (2-HO-ASE) binding site.

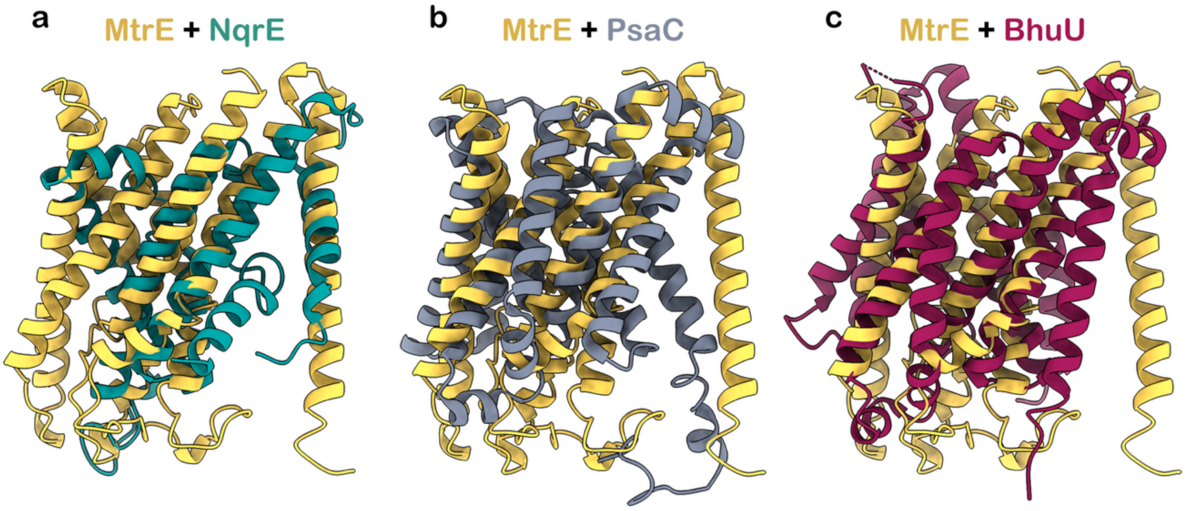

Supplementary Fig. 5. Structural comparison of MtrE with representative membrane transport proteins identified by DALI. a, MtrE superimposed with the sodium-pumping subunit NqrE of the NADH-ubiquinone oxidoreductase (Nqr complex) from *Vibrio cholerae* (PDB: 7xk3-E, RMSD = 4.1 Å). **b**, MtrE superimposed with the membrane subunit PsaC of the manganese importer (PsaBC complex) from *Streptococcus pneumoniae* (PDB: 7kyp-F, RMSD = 5.5 Å). **c**, MtrE with the membrane subunit BhuU of the ABC heme-importer (BhuUV complex) from *Burkholderia cenocepacia* (PDB: 5B58-A).

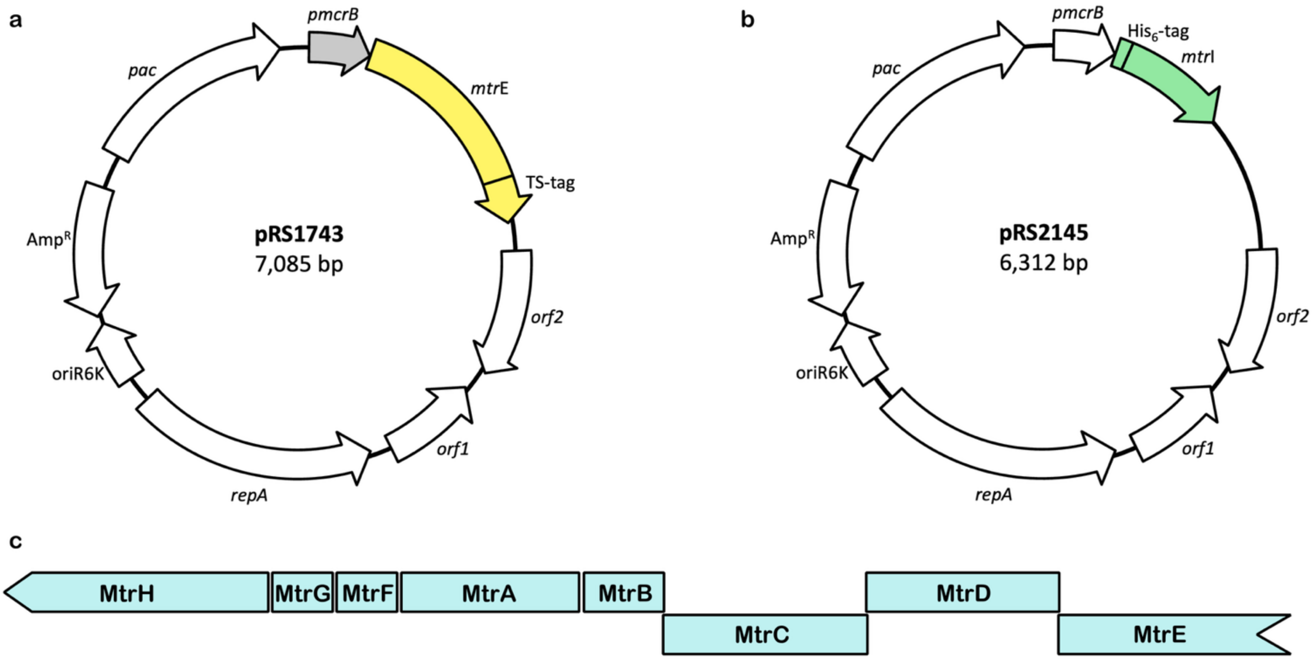

d

MKCEACGRESDTKYCNDCGKVMDEVVRRVGEARWAAIDDCSFIYPLVQRVGRGEATVNDIIQALDVED

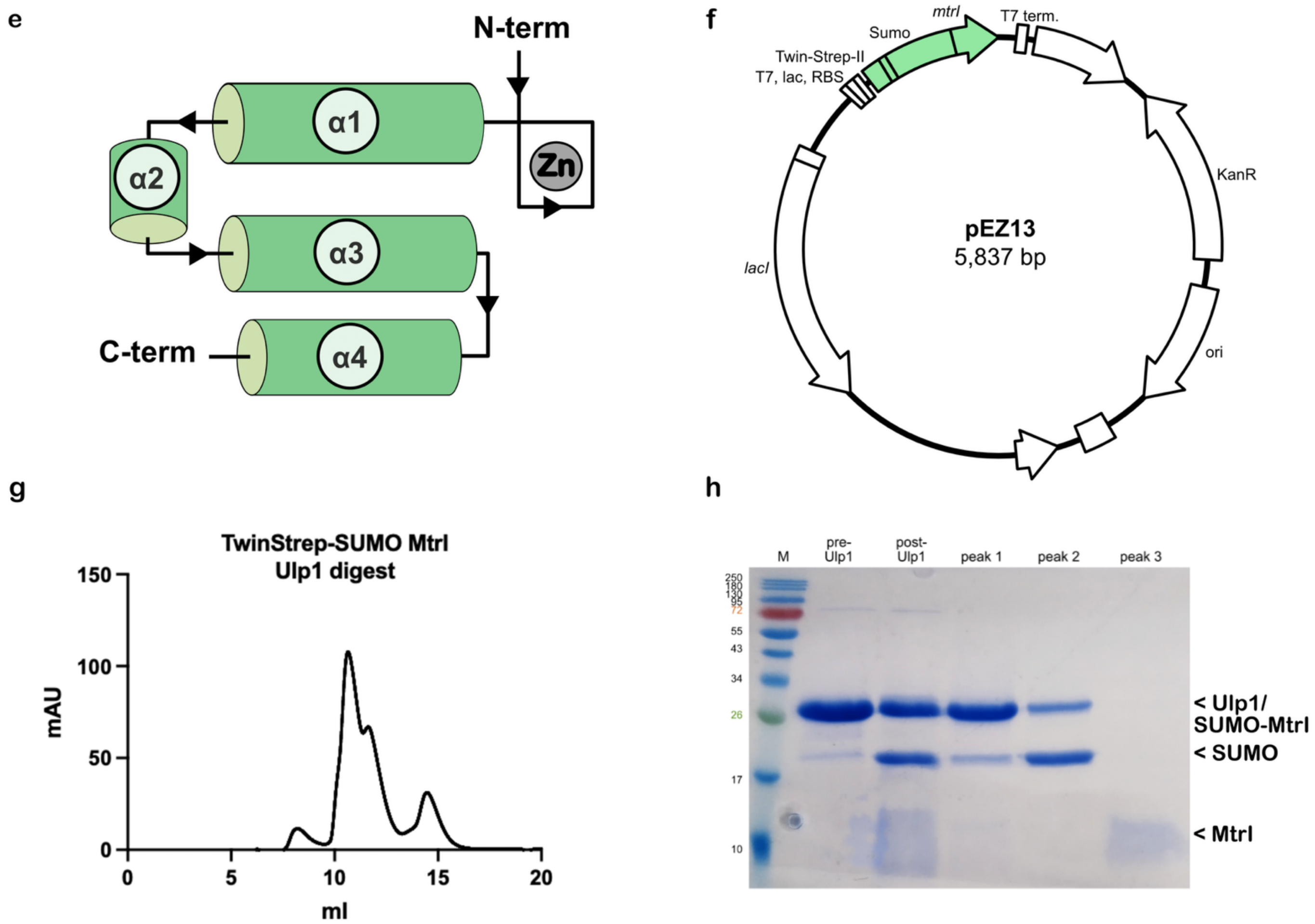

i

Sample	^{56}Fe / Protein	±^{56}Fe/Protein	^{59}Co / Protein	±^{59}Co/Protein	^{66}Zn / Protein	±^{66}Zn/Protein
MtrI_SEC_peak3	<0	0,02	0,00	0,00	0,90	0,04

Supplementary Fig. 6. Plasmids and additional information on MtrI. a, Map of plasmid pRS1743 used for expression of MtrE with a C-terminal TS-tag in *M. mazei*. Shown in white are the pRS1595 backbone elements (Thomsen and Schmitz 2022[1]*ƒ*), in grey the *pmcr*B promotor and in yellow the *mtr*E-gene with TS-tag. **b**, Map of plasmid pRS2145 used for expression of MtrI with a N-terminal His_6-tag in *M. mazei*. Shown in white are the pRS1807 backbone elements (Hüttermann and Schmitz 2024[2]) and in green the *mtrI*-gene and His_6-tag. **c**, Operon structure of Mtr in *M. mazei*. **d,** protein sequence of MM_2401 (MtrI). **e,** Secondary structure topology of MtrI. **f,** Map of plasmid pEZ13 used for recombinant expression of TS-SUMO-MtrI in *E. coli* BL21. **g**, Size-exclusion-chromatography of concentrated StrepTactin-eluates of TS-SUMO-MtrI on a Superdex 75 Increase 10/300 column. Double-peak at around 10-12 ml corresponds to TS-SUMO-MtrI and TS-SUMO. Peak at around 14.5 ml corresponds to MtrI cleavage product. **h**, SDS-gel of the TS-SUMO-MtrI purification. Lanes from left to right: Protein ladder, StrepTactin-eluate before Ulp1-digest, StrepTactin-eluate after Ulp1-digest, SEC-peak at 10.7 ml, SEC-peak at 11.7 ml, SEC peak at 14.5 ml. **I**, ICP-MS of purified MtrI measured as technical triplicates. Values are calculated based on a predicted molecular weight of 7.62 kDa.

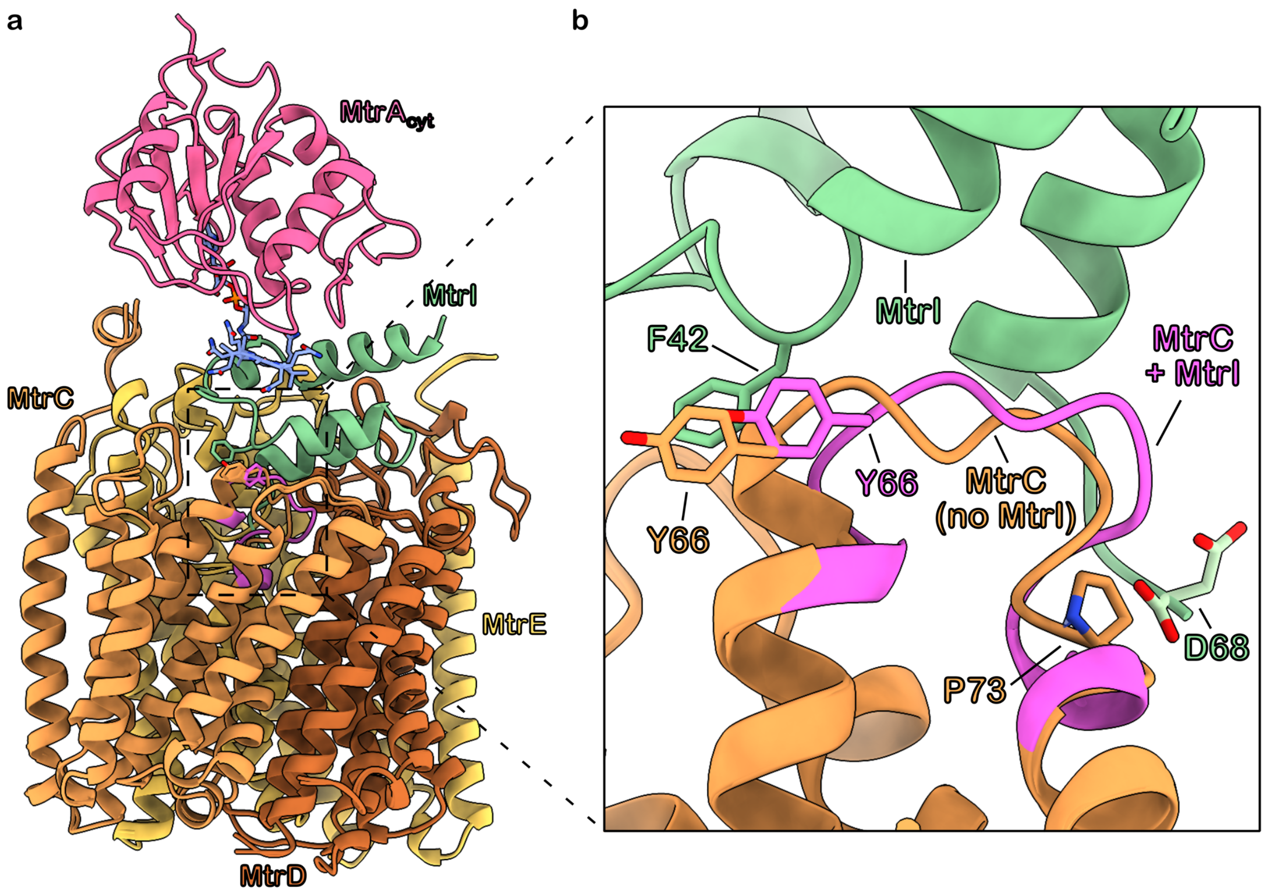

Supplementary Fig. 7. Structural comparison of MtrCDE with and without MtrI bound. a, Superimposition of MtrCDE with MtrI and MtrA together with MtrCDE without MtrI bound shows overall high similarity. **b,** Zoom-in on the superimposed MtrC loop that shows MtrI accommodating structural differences. Loop-residues of MtrC (63-75) interacting with MtrI are colored purple. Interacting residues and C-terminus of MtrI shown in sticks.

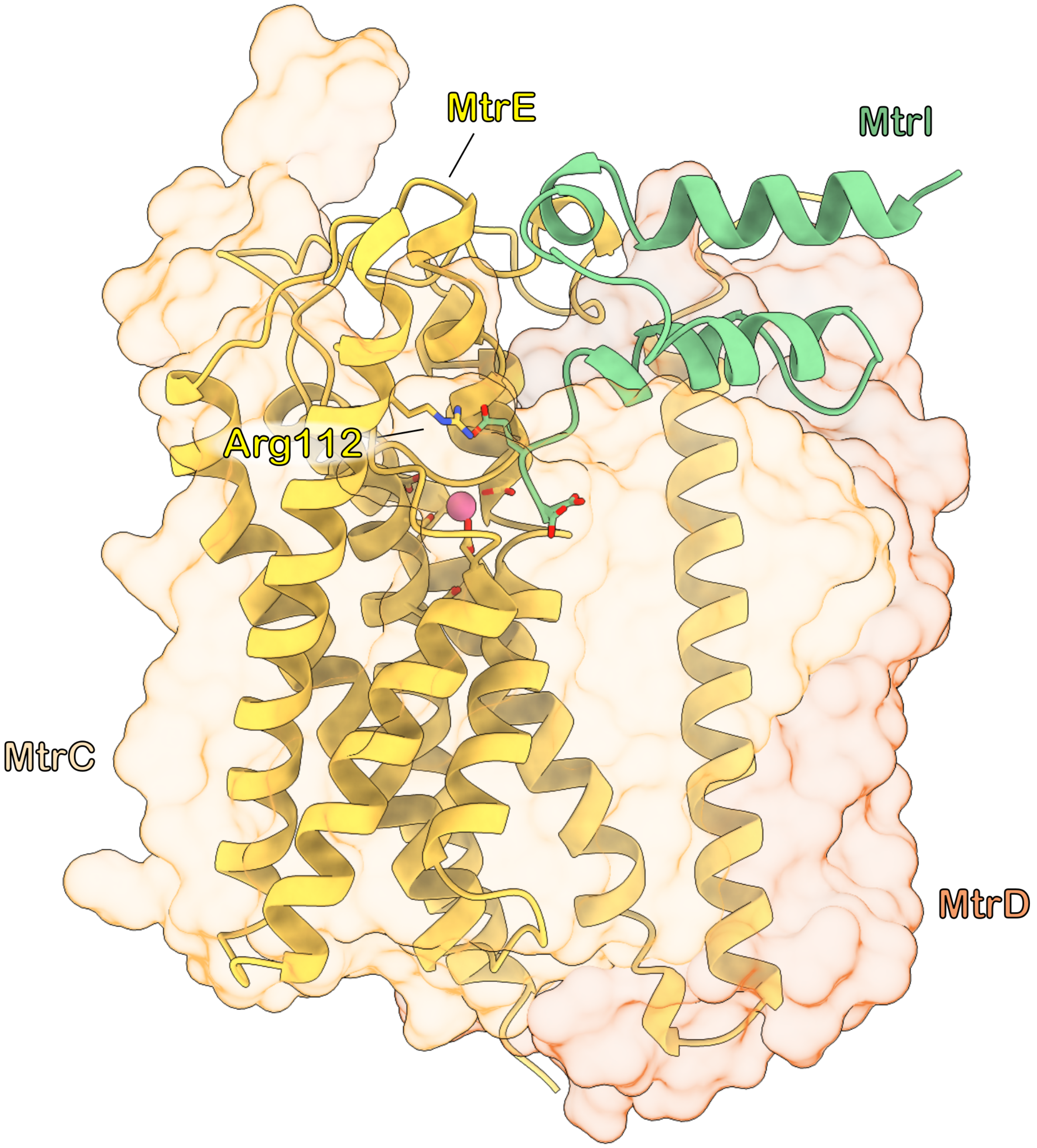

Supplementary Fig. 8. The C-terminus of MtrI extends toward the sodium pocket and interacts with Arg112 located within the cavity. The sodium ion is shown as a pink sphere.

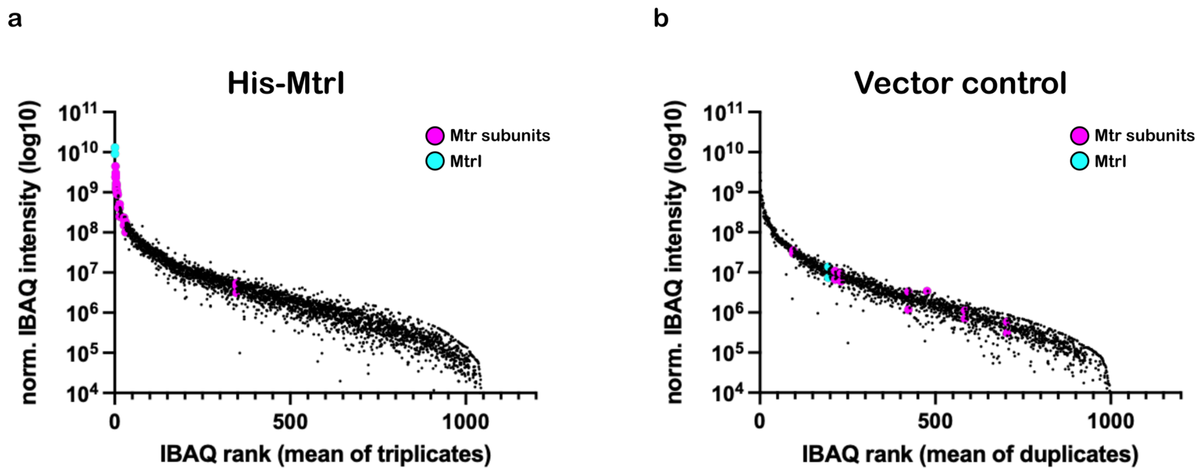

Supplementary Fig. 9. Label-free quantification of Mtr subunits by His$_6$-MtrI pulldown. **a**, Mass spectrometry analysis of Ni-NTA purification from *M. mazei* expressing His$_6$-tagged MtrI. Log$_{10}$(iBAQ) values of proteins (biological triplicates, calculated with MaxQuant v.2.6.5.0) are plotted against their rank position (mean of triplicates, n = 3). **b**, Equivalent analysis from an empty-vector *M. mazei* control strain shown as log$_{10}$(iBAQ) values (biological duplicates, n = 2) plotted against their rank position. **a, b**, His$_6$-MtrI pulldown leads to clear enrichment of Mtr subunits compared to the empty-vector control. The reciprocal pulldown of Mtr subunits confirms the stable association of MtrI with the complex.

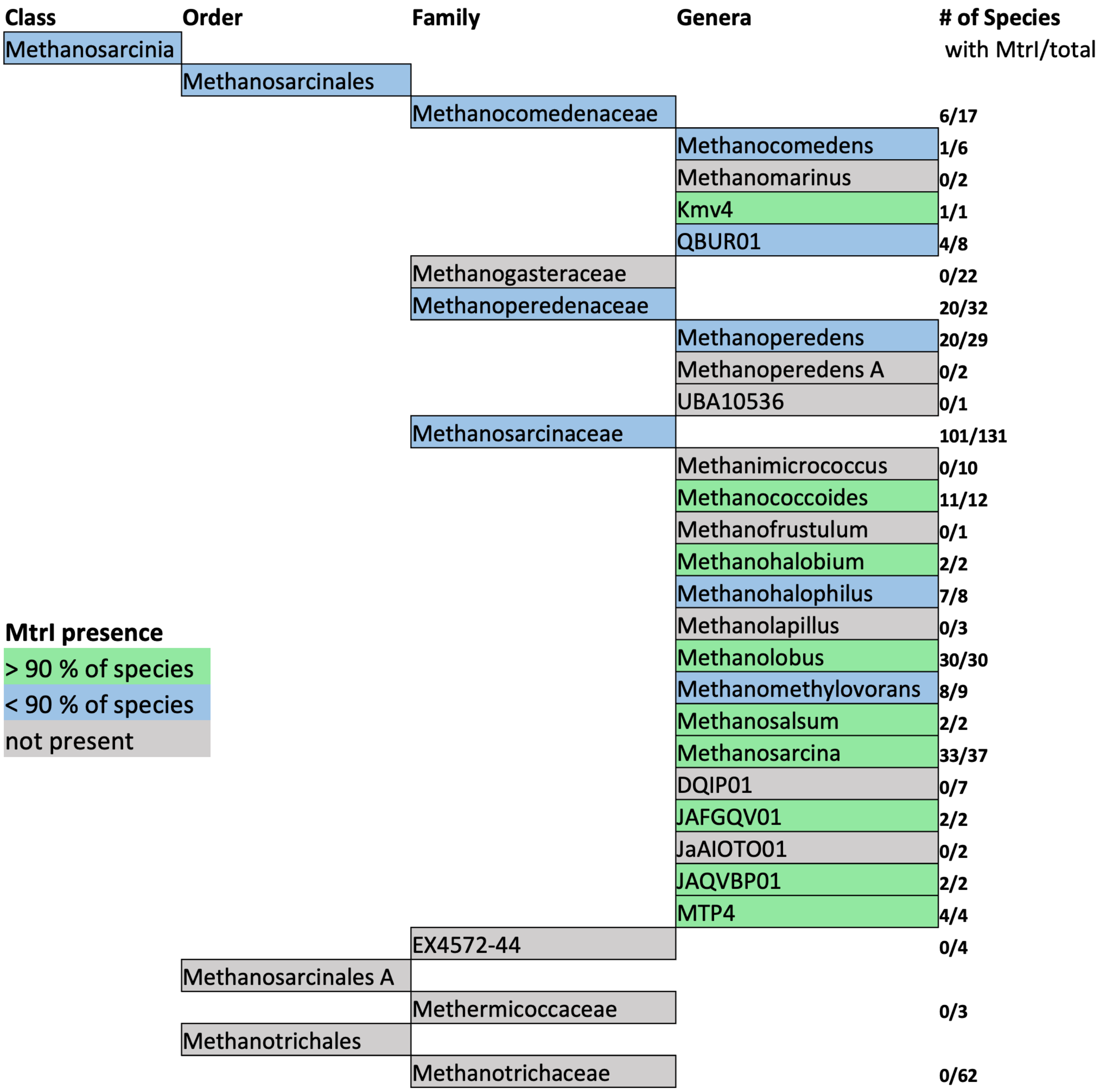

Supplementary Fig. 10. Presence of MtrI across the different Methanosarcinia clades based on tblastn. MtrI absence I shown in grey, presence in less than 90% of the species in grey-blue and presence in more than 90% of the species in green. The taxonomic tree was drawn based on the genome taxonomy database (Parks et al. 2022[3], accessed 26th of August 2025).

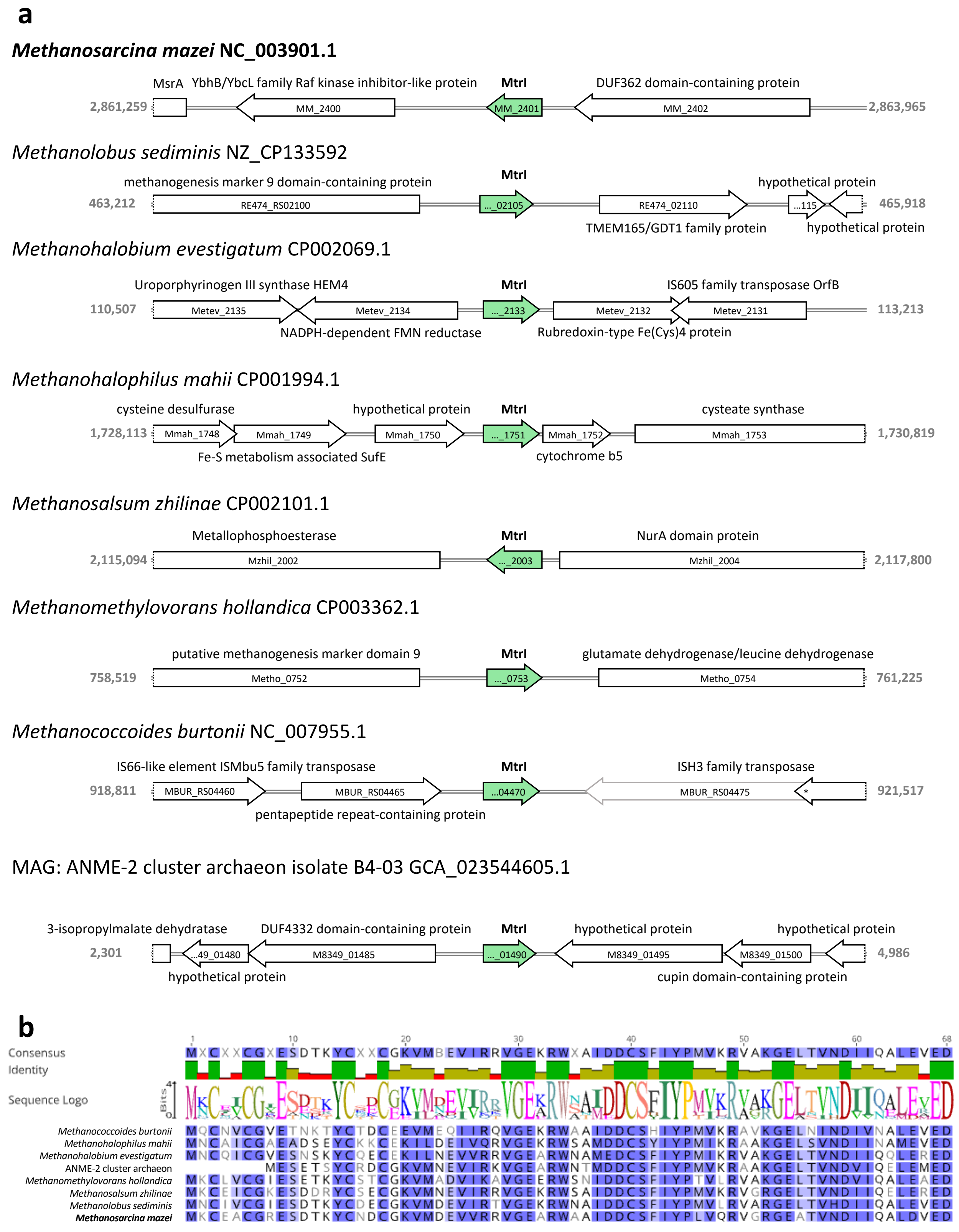

Supplementary Fig. 11: MtrI genome localization (a) and amino acid sequence alignment (b) of representatives from different genera.

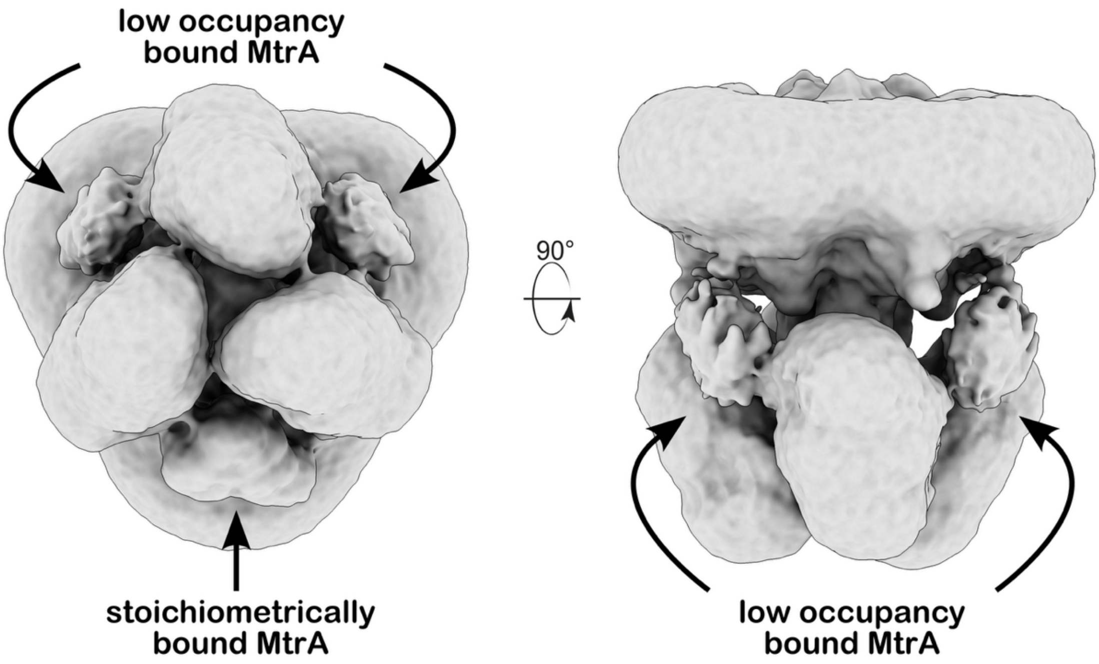

Supplementary Fig. 12: Low occupancy presence of MtrA bound to MtrI at all MtrCDE sites becomes apparent by viewing the consensus map at low threshold (0.0045) and filtering of the map in ChimeraX using gaussian filtering with a width of 1.70.

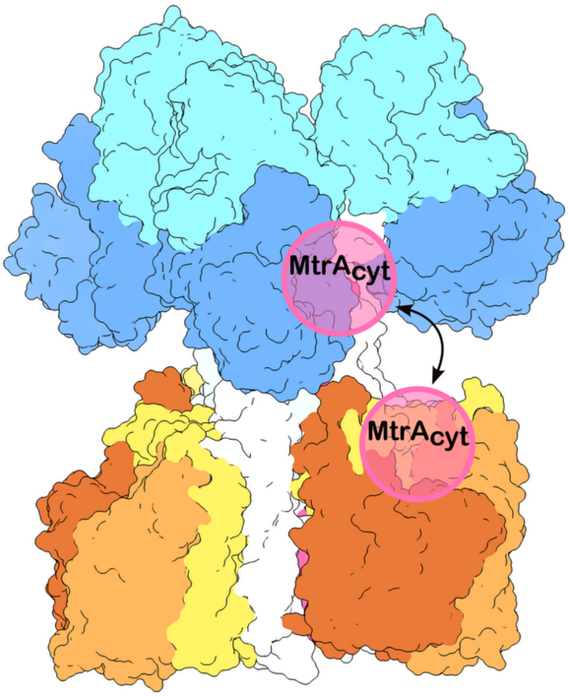

Supplementary Fig. 13: Potential states of cytosolic MtrA. $MtrA_{cyt}$ likely moves between the proximal MtrH and the MtrCDE subcomplex.

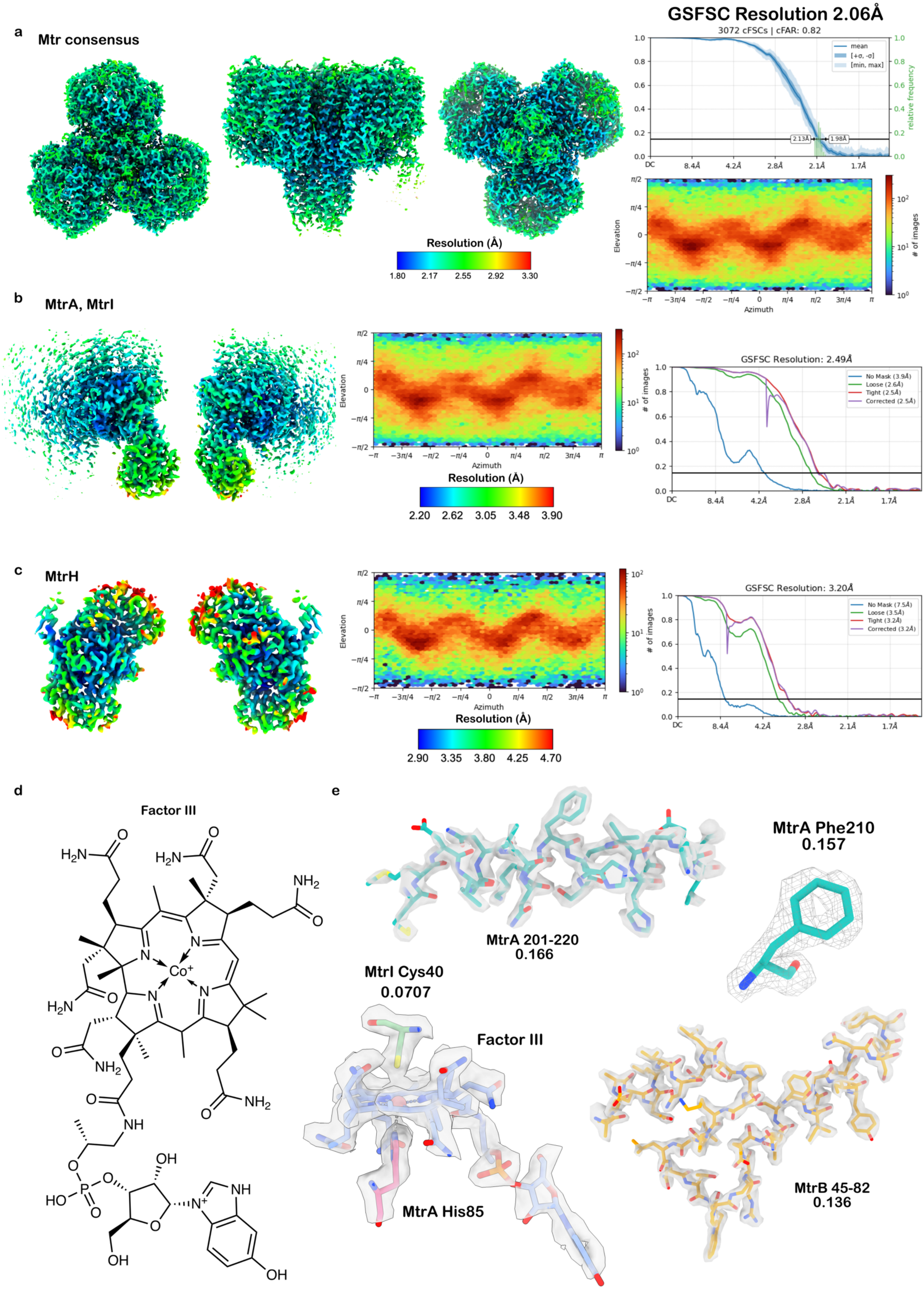
a
Mtr consensus
GSFSC Resolution 2.06Å
3072 cFSCs | cFAR: 0.82
mean
[+σ, -σ]
[min, max]
relative frequency
2.13Å
1.98Å
DC
8.4Å
4.2Å
2.8Å
2.1Å
1.7Å
Elevation
Azimuth
# of images
Resolution (Å)
1.80
2.17
2.55
2.92
3.30
b
MtrA, MtrI
Elevation
Azimuth
# of images
Resolution (Å)
2.20
2.62
3.05
3.48
3.90
GSFSC Resolution: 2.49Å
No Mask (3.9Å)
Loose (2.6Å)
Tight (2.5Å)
Corrected (2.5Å)
c
MtrH
Elevation
Azimuth
# of images
Resolution (Å)
2.90
3.35
3.80
4.25
4.70
GSFSC Resolution: 3.20Å
No Mask (7.5Å)
Loose (3.5Å)
Tight (3.2Å)
Corrected (3.2Å)
d
Factor III
e
MtrA Phe210
0.157
MtrA 201-220
0.166
MtrI Cys40
0.0707
Factor III
MtrA His85
MtrB 45-82
0.136

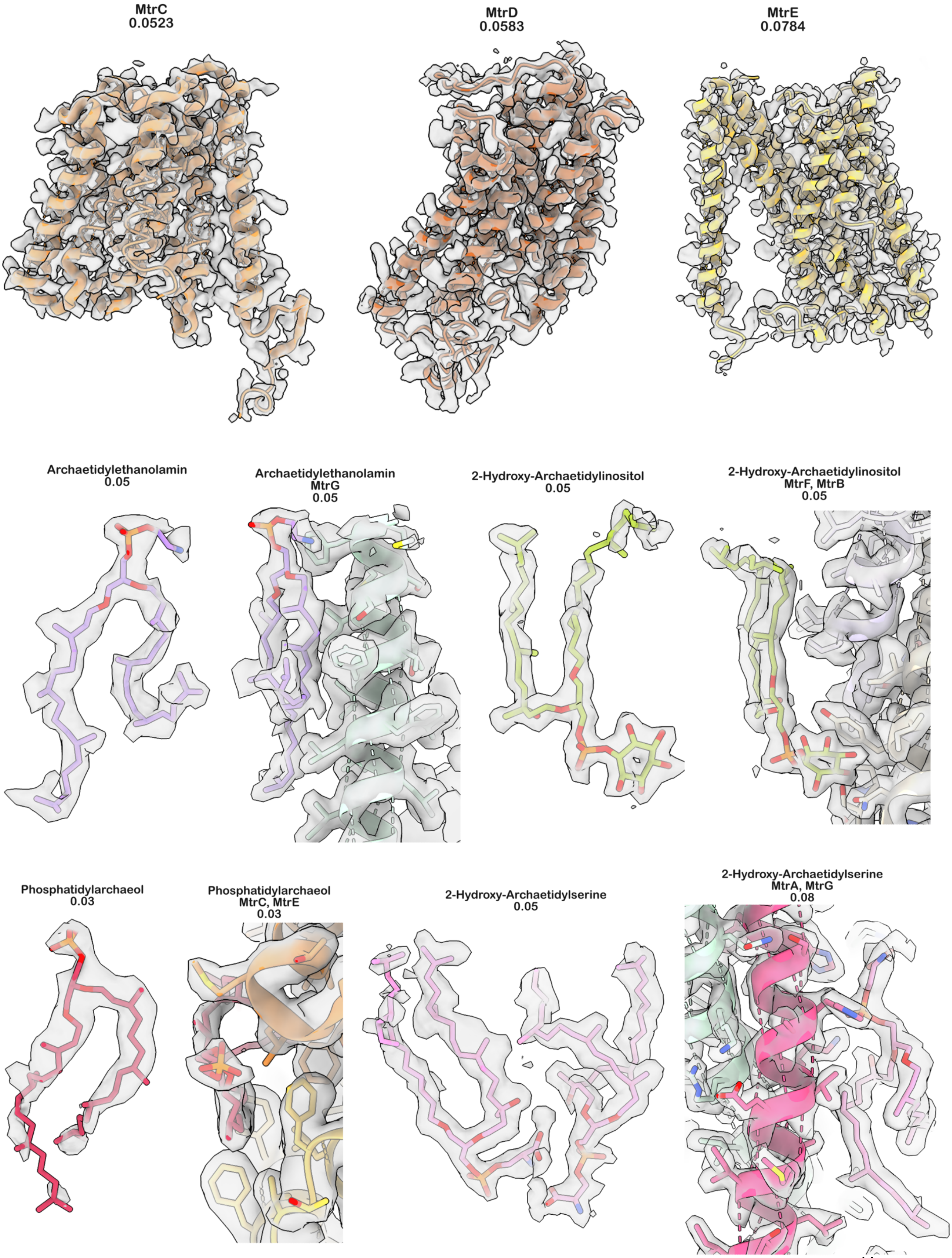
MtrC
0.0523
MtrD
0.0583
MtrE
0.0784
Archaetidylethanolamin
0.05
Archaetidylethanolamin
MtrG
0.05
2-Hydroxy-Archaetidylinositol
0.05
2-Hydroxy-Archaetidylinositol
MtrF, MtrB
0.05
Phosphatidylarchaeol
0.03
Phosphatidylarchaeol
MtrC, MtrE
0.03
2-Hydroxy-Archaetidylserine
0.05
2-Hydroxy-Archaetidylserine
MtrA, MtrG
0.08

Supplementary Fig. 14. Local resolution estimates and quality of cryo-EM-maps. **a**-**c**, Cryo-EM maps of Mtr consensus (core), locally refined MtrA and locally refined MtrH colored by local resolution values estimated in cryoSPARC**.** The angular distribution of particle orientations used in the final reconstruction is shown. Gold standard Fourier shell correlation (GSFSC) plots show resolutions at a 0.143 threshold (black line). **d**, Structural formula of Factor III. **e**, Representative regions of the Cryo-EM maps shown at corresponding threshold levels together with atomic models displayed as sticks or cartoon demonstrate the high quality of the maps.

Supplementary Table 1, CryoEM data collection, refinement and validation statistics

	**Mtr Composite map** (EMDB-53361) (PDB: 9QTS)	**MtrA local map** (EMDB-53360) (PDB:9QTR)	**Mtr consensus map** (EMDB-53359) (PDB:9QTQ)	**MtrH local map** (EMDB-53358) (PDB:9QTP)
**Data collection and processing**				
Magnification	165 000x	165 000x	165 000x	165 000x
Voltage (kV)	300	300	300	300
Electron exposure (e–/Å^2)	60	60	60	60
Defocus range (μm)	0.5-2	0.5-2	0.5-2	0.5-2
Pixel size (Å)	0.73	0.73	0.73	0.73
Symmetry imposed	C1	C1	C1	C1
Initial particle images (no.)	1 096 091	1 096 091	1 096 091	1 096 091
Final particle images (no.)	174 856	136 531	136 531	56 229
Map resolution (Å)		2.49	2.49	2.33
FSC threshold		0.143	0.143	0.143
Map resolution range (Å)		2.2-3.2	1.8-2.8	2.9-3.8
**Refinement**				
Initial model used (PDB code)	*de novo,* ModelAngelo, AlphaFold	*de novo,* ModelAngelo, *AlphaFold*	*de novo,* ModelAngelo, *AlphaFold*	De novo, AlphaFold
Model resolution (Å)		2.8	2.1	3.3
FSC threshold		0.5	0.5	0.5
Model resolution range (Å)		2.4-2.8	2.0-2.1	3.1-3.3
Map sharpening *B* factor (Å^2)		58	38.3	62.6
Model composition				
Non-hydrogen atoms	45429	1698	28656	5082
Protein residues	5432	215	3231	670
Ligands	19	1	18	0
B factors (Å^2)				
Protein	42.58	31.70	29.12	75.51
Ligand	53.32	14.60	35.59	
R.m.s. deviations				
Bond lengths (Å)	0.002	0.003	0.005	0.003
Bond angles (°)	0.456	0.676	0.634	0.563
Validation				
MolProbity score	2.13	2.65	1.83	2.06
Clashscore	22.44	9.16	13.81	7.59
Poor rotamers (%)	1.46	6.98	1.66	2.57
Ramachandran plot				
Favored (%)	97.08	91.00	97.93	95.17
Allowed (%)	2.87	8.53	2.04	4.68
Disallowed (%)	0.04	0.47	0.03	0.15

1. Thomsen, J. & Schmitz, R. A. Generating a Small Shuttle Vector for Effective Genetic Engineering of Methanosarcina mazei Allowed First Insights in Plasmid Replication Mechanism in the Methanoarchaeon. *Int J Mol Sci* **23**, 11910 (2022).
2. Hüttermann, J. & Schmitz, R. Compiling a versatile toolbox for inducible gene expression in Methanosarcina mazei. *microLife* **5**, uqae019 (2024).
3. Parks, D. H. *et al.* GTDB: an ongoing census of bacterial and archaeal diversity through a phylogenetically consistent, rank normalized and complete genome-based taxonomy. *Nucleic Acids Res* **50**, D785–D794 (2022).
