# Supplementary material for: Structure of the Methanosarcina mazei Mtr complex bound to the oxygen-stress responsive small protein MtrI
Source: Nat Commun. 2025 Dec 23;17:133. doi: 10.1038/s41467-025-67705-5 (PMC12774963; doi:10.1038/s41467-025-67705-5)
Supplement: Supplementary file 2 — Reporting Summary [file 41467_2025_67705_MOESM2_ESM.pdf]

nature portfolio

Corresponding author(s): Jan M. Schuller
Ruth A. Schmitz

Last updated by author(s): Nov 27, 2025

Reporting Summary

Nature Portfolio wishes to improve the reproducibility of the work that we publish. This form provides structure for consistency and transparency in reporting. For further information on Nature Portfolio policies, see our Editorial Policies and the Editorial Policy Checklist.

Statistics

For all statistical analyses, confirm that the following items are present in the figure legend, table legend, main text, or Methods section.

n/a	Confirmed	
☐	☒	The exact sample size (*n*) for each experimental group/condition, given as a discrete number and unit of measurement
☐	☒	A statement on whether measurements were taken from distinct samples or whether the same sample was measured repeatedly
☐	☒	The statistical test(s) used AND whether they are one- or two-sided<br>*Only common tests should be described solely by name; describe more complex techniques in the Methods section.*
☐	☒	A description of all covariates tested
☒	☐	A description of any assumptions or corrections, such as tests of normality and adjustment for multiple comparisons
☐	☒	A full description of the statistical parameters including central tendency (e.g. means) or other basic estimates (e.g. regression coefficient) AND variation (e.g. standard deviation) or associated estimates of uncertainty (e.g. confidence intervals)
☐	☒	For null hypothesis testing, the test statistic (e.g. *F*, *t*, *r*) with confidence intervals, effect sizes, degrees of freedom and *P* value noted<br>*Give P values as exact values whenever suitable.*
☒	☐	For Bayesian analysis, information on the choice of priors and Markov chain Monte Carlo settings
☒	☐	For hierarchical and complex designs, identification of the appropriate level for tests and full reporting of outcomes
☒	☐	Estimates of effect sizes (e.g. Cohen's *d*, Pearson's *r*), indicating how they were calculated

Our web collection on statistics for biologists contains articles on many of the points above.

Software and code

Policy information about availability of computer code

Data collection	Smart EPU 2.14 software for automated cryo-EM data acquisition on Titan Krios. Qtegra v2.18 for ICP-MS (Thermo Fisher scientific), Thermo Fisher Xcalibur (v.4.4) for protein mass-spectrometry data acquisition. UNICORN v.7 (cytiva) for chromatography, AcquireMP v.2.3 (Refeyn) for mass photometry data acquisition.
Data analysis	CryoSPARC v4.6.1–v4.7.1; for cryo-EM data processing, Coot (v.0.9.8.91) for model building, AceDRG (from CCP4Interface 8.0.015) and eLBOW (from Phenix v.1.21-5207) for ligand cif-file generation, ChimeraX (v.1.8) for model building and visualisation, ModelAngelo v.1.0 and AlphaFold2 for structure prediction, PHENIX v.1.21-5207 for model building and refinement, Qtegra v2.18, Proteome Discoverer v.1.4 (Thermo Fisher) protein mass spectrometry search, MaxQuant v.2.6.5.0 for analysis and quantification of mass-spectrometry, GraphPad Prism v.10 for plotting and statistical analysis, DiscoverMP v.2.3 (Refeyn) for mass photometry data processing. ChemDraw 21.0.0 for drawing of structural formulas. Genomic data were retrieved using NCBI datasets command-line tools v.16.22.1. Genome quality and taxonomy were assessed using CheckM2 v1.0.253 and GTDB-Tk v2.4.0. ORF searches were performed using tblastn v2.13.0+.

For manuscripts utilizing custom algorithms or software that are central to the research but not yet described in published literature, software must be made available to editors and reviewers. We strongly encourage code deposition in a community repository (e.g. GitHub). See the Nature Portfolio guidelines for submitting code & software for further information.

Data

Policy information about availability of data

All manuscripts must include a data availability statement. This statement should provide the following information, where applicable:

- Accession codes, unique identifiers, or web links for publicly available datasets
- A description of any restrictions on data availability
- For clinical datasets or third party data, please ensure that the statement adheres to our policy

Data availability
Several cryo-EM maps have been deposited in the Electron Microscopy Data Bank (EMDB) under following accession codes. Composite map: EMD-53361, Consensus map: EMD-53359, MtrA local refinement: EMD-53360, MtrH local refinement: EMD-53358. Corresponding coordinate files have been deposited in the RCSB Protein Data Bank (PDB) under following accession codes: 9QTS, 9QTQ, 9QTR, 9QTP.
The mass spectrometry proteomics data have been deposited to the ProteomeXchange Consortium (http://proteomecentral.proteomexchange.org) via the PRIDE partner repository (Vizcaíno et al., 2013) with the dataset identifier < PXD064689>.

Research involving human participants, their data, or biological material

Policy information about studies with human participants or human data. See also policy information about sex, gender (identity/presentation), and sexual orientation and race, ethnicity and racism.

Reporting on sex and gender	*Use the terms sex (biological attribute) and gender (shaped by social and cultural circumstances) carefully in order to avoid confusing both terms. Indicate if findings apply to only one sex or gender; describe whether sex and gender were considered in study design; whether sex and/or gender was determined based on self-reporting or assigned and methods used. Provide in the source data disaggregated sex and gender data, where this information has been collected, and if consent has been obtained for sharing of individual-level data; provide overall numbers in this Reporting Summary. Please state if this information has not been collected. Report sex- and gender-based analyses where performed, justify reasons for lack of sex- and gender-based analysis.*
Reporting on race, ethnicity, or other socially relevant groupings	*Please specify the socially constructed or socially relevant categorization variable(s) used in your manuscript and explain why they were used. Please note that such variables should not be used as proxies for other socially constructed/relevant variables (for example, race or ethnicity should not be used as a proxy for socioeconomic status). Provide clear definitions of the relevant terms used, how they were provided (by the participants/respondents, the researchers, or third parties), and the method(s) used to classify people into the different categories (e.g. self-report, census or administrative data, social media data, etc.) Please provide details about how you controlled for confounding variables in your analyses.*
Population characteristics	*Describe the covariate-relevant population characteristics of the human research participants (e.g. age, genotypic information, past and current diagnosis and treatment categories). If you filled out the behavioural & social sciences study design questions and have nothing to add here, write "See above."*
Recruitment	*Describe how participants were recruited. Outline any potential self-selection bias or other biases that may be present and how these are likely to impact results.*
Ethics oversight	*Identify the organization(s) that approved the study protocol.*

Note that full information on the approval of the study protocol must also be provided in the manuscript.

Field-specific reporting

Please select the one below that is the best fit for your research. If you are not sure, read the appropriate sections before making your selection.

[x] Life sciences [ ] Behavioural & social sciences [ ] Ecological, evolutionary & environmental sciences

For a reference copy of the document with all sections, see nature.com/documents/nr-reporting-summary-flat.pdf

Life sciences study design

All studies must disclose on these points even when the disclosure is negative.

Sample size	Sample size was not predetermined by statistical methods but was chosen based on a downscaling of cryo-EM sample preparation that allowed reproducible purification. For each replicate, 0.75–1 L of Methanosarcina mazei culture was used. This volume provided sufficient protein for quantitative mass spectrometry analysis while maintaining consistency across replicates. For cryo-EM, over 20,000 micrographs were acquired to obtain enough Mtr particles, and the subsequent 3D reconstruction reached 2.1 Å resolution, allowing modeling at the residue level.
Data exclusions	No data were excluded from any analyses. All measurements from mass spectrometry and cryo-EM experiments were included in the reported results. For cryo-EM, particle selection and classification were performed as described in the Methods, and only particles failing standard quality thresholds during automated processing were removed.

Replication	For experiments testing the effect of oxygen on MtrI association with the complex, measurements were performed in biological triplicates. His-MtrI pulldown experiments in M. mazei were performed in biological triplicates, while the empty plasmid control was performed in biological duplicates. Attempts to replicate the results were successful.
Randomization	Not applicable. All experiments were performed on identical biological samples, and comparisons were made between paired measurements.
Blinding	Not applicable. Measurements were quantitative and automated (mass spectrometry), minimizing operator bias.

Reporting for specific materials, systems and methods

We require information from authors about some types of materials, experimental systems and methods used in many studies. Here, indicate whether each material, system or method listed is relevant to your study. If you are not sure if a list item applies to your research, read the appropriate section before selecting a response.

Materials & experimental systems

n/a	Involved in the study
☐	☒ Antibodies
☒	☐ Eukaryotic cell lines
☒	☐ Palaeontology and archaeology
☒	☐ Animals and other organisms
☒	☐ Clinical data
☒	☐ Dual use research of concern
☒	☐ Plants

Methods

n/a	Involved in the study
☒	☐ ChIP-seq
☒	☐ Flow cytometry
☒	☐ MRI-based neuroimaging

Antibodies

Antibodies used	Horseradish peroxidase (HRP)-coupled anti-TwinStrep-tag antibody (IBA Lifesciences, cat. no. 2-1509-001) at a dilution of 1:5,000
Validation	The HRP-coupled anti-TwinStrep-tag antibody (IBA Lifesciences, cat. no. 2-1509-001) is a tag-specific antibody that recognizes the engineered Twin-Strep peptide sequence and is therefore species-independent. The manufacturer validates this antibody for Western blotting of TwinStrep-tagged proteins. In this study, the antibody was further validated by detecting a single band at the expected molecular weight for TS-tagged MtrE in purified samples (Supp, Fig. 1b), consistent with the expected specificity. No additional antibodies were used in this manuscript.

Plants

Seed stocks	*Report on the source of all seed stocks or other plant material used. If applicable, state the seed stock centre and catalogue number. If plant specimens were collected from the field, describe the collection location, date and sampling procedures.*
Novel plant genotypes	*Describe the methods by which all novel plant genotypes were produced. This includes those generated by transgenic approaches, gene editing, chemical/radiation-based mutagenesis and hybridization. For transgenic lines, describe the transformation method, the number of independent lines analyzed and the generation upon which experiments were performed. For gene-edited lines, describe the editor used, the endogenous sequence targeted for editing, the targeting guide RNA sequence (if applicable) and how the editor was applied.*
Authentication	*Describe any authentication procedures for each seed stock used or novel genotype generated. Describe any experiments used to assess the effect of a mutation and, where applicable, how potential secondary effects (e.g. second site T-DNA insertions, mosiacism, off-target gene editing) were examined.*
