# Supplementary material for: Structure of the Methanosarcina mazei Mtr complex bound to the oxygen-stress responsive small protein MtrI
Source: Nat Commun. 2025 Dec 23;17:133. doi: 10.1038/s41467-025-67705-5 (PMC12774963; doi:10.1038/s41467-025-67705-5)
Supplement: Supplementary file 3 — Description of Additional Supplementary Files [file 41467_2025_67705_MOESM3_ESM.pdf]

Description of Additional Supplementary Files

Supplementary Data 1. DALI structural similarity search of MtrE

This text file contains the PDB50 output of a DALI search using the *M. mazei* MtrE structure as the query. The file lists all identified structural homologs, including Z-scores, RMSDs, and %id.

Supplementary Data 2. BLAST searches for MtrI homologs

This excel file contains results different BLAST methods used for MtrI homology search. It contains worksheets with results of tBLASTn search of MtrI against locally downloaded Euryarchaeota genomes as well as webbased BLASTn, BLASTp and tBLASTn searches of MtrI against all available genomes.

Supplementary Data 3. Structural similarity searches for MtrI

This Excel file contains the results of structural homology searches for MtrI using Foldseek and the tabular output of DALI. Individual worksheets contain the ranked outputs from each tool.
